# Harmonization Strategies in Multicenter MRI-Based Radiomics

**DOI:** 10.3390/jimaging8110303

**Published:** 2022-11-07

**Authors:** Elisavet Stamoulou, Constantinos Spanakis, Georgios C. Manikis, Georgia Karanasiou, Grigoris Grigoriadis, Theodoros Foukakis, Manolis Tsiknakis, Dimitrios I. Fotiadis, Kostas Marias

**Affiliations:** 1 Computational BioMedicine Laboratory (CBML), Foundation for Research and Technology—Hellas (FORTH), 700 13 Heraklion, Greece; 2 Department of Oncology-Pathology, Karolinska Institutet, 171 77 Stockholm, Sweden; 3 Unit of Medical Technology and Intelligent Information Systems, Department of Materials Science and Engineering, University of Ioannina, 451 10 Ioannina, Greece; 4 Department of Electrical & Computer Engineering, Hellenic Mediterranean University, 714 10 Heraklion, Greece; 5 Department of Biomedical Research, Institute of Molecular Biology and Biotechnology—FORTH, University Campus of Ioannina, 451 15 Ioannina, Greece

**Keywords:** radiomics, MRI, batch effect, variability, feature stability, image harmonization, feature harmonization, multicenter studies, standardization

## Abstract

Radiomics analysis is a powerful tool aiming to provide diagnostic and prognostic patient information directly from images that are decoded into handcrafted features, comprising descriptors of shape, size and textural patterns. Although radiomics is gaining momentum since it holds great promise for accelerating digital diagnostics, it is susceptible to bias and variation due to numerous inter-patient factors (e.g., patient age and gender) as well as inter-scanner ones (different protocol acquisition depending on the scanner center). A variety of image and feature based harmonization methods has been developed to compensate for these effects; however, to the best of our knowledge, none of these techniques has been established as the most effective in the analysis pipeline so far. To this end, this review provides an overview of the challenges in optimizing radiomics analysis, and a concise summary of the most relevant harmonization techniques, aiming to provide a thorough guide to the radiomics harmonization process.

## 1. Introduction

**Figure 1:**
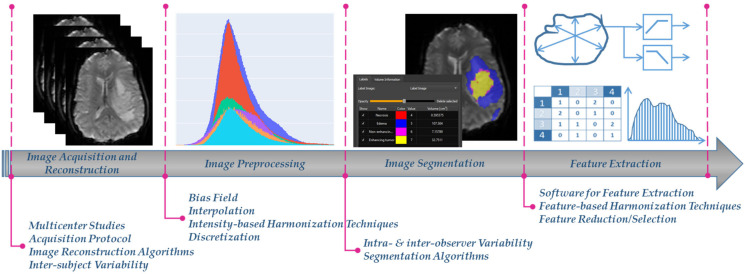
The radiomics analysis workflow, as partially depicted in one of our radiomics studies [5], and the different factors that affect harmonization.

Radiomics, a rapidly evolving field in medical image analysis, aims to enable digital decoding of images into high-throughput quantitative features, aiding earlier and more precise care in clinical decision making [1,2]. It is applied in different medical image modalities, including magnetic resonance imaging (MRI), computed tomography (CT) and positron emission tomography (PET) based on the concept that images potentially contain hidden patterns of the underlying pathophysiology of the examined tissue [1,2]. The extracted image features are usually volume, shape, intensity or textural features, calculated using several mathematical equations from the image analysis domain [3]. Radiomics features are prone to bias and variability caused by a number of factors such as the: (i) acquisition protocols and image reconstruction settings, (ii) image preprocessing, (iii) detection and segmentation of the examined region of interest (ROI), and (iv) radiomics feature extraction (Figure 1) [4]. This can significantly influence the absolute value and the statistical distribution of the radiomics features, subsequently having a considerable impact on the robustness and generalizability of any further analysis derived from them.

In a significant number of radiomics studies, the above variability effects are significantly reduced when images are obtained from a single center or a unique acquisition protocol/vendor [6,7,8,9,10,11,12]. Nevertheless, this homogeneity in the radiomics features yields to analyses that are susceptible to center and protocol/vendor specific dependencies, thus are not diverse enough to generalize to new images obtained from a different data source or acquisition setup. To address this issue, multicenter radiomics studies can foster radiomics transfer and applicability in the clinical setting [13]. However, although studies using data from multiple centers potentially accelerate radiomics generalizability, clinical adoption of radiomics is limited by variability issues that exist in all analysis steps (Figure 1) [14].

Harmonization processes have been proposed as a solution to reduce the inherent variability in medical images. In general, harmonization attempts to overcome the lack of comparability between medical images and the absence of reproducibility in radiomics features. For example, it is often difficult to clarify whether the results of a study can be applied to data obtained at another institution or whether results from multiple institutions can be pooled together. In other words, harmonization aims towards consistent and robust findings in multicenter radiomics studies. To the best of our knowledge, up to now, harmonization has been investigated less extensively in MRI compared to PET and CT [2]. This might be due to the technical challenges of the MRI, comprising among others non-standardized signal intensities that are highly dependent on the manufacturer and image acquisition specifics (e.g., scanner model, coil, sequence type, acceleration, bandwidth, field echo, matrix size, voxel size, slice thickness, slice gap, repetition time, echo time, and acquisition time) [15,16]. Indicatively, findings of a recent study on brain MRI showed that the calculated radiomics features vary widely depending on the pulse sequence, while another study on cervical cancer demonstrated that only few radiomics features were robust across different MRI scanners and acquisition parameters [17,18]. For these reasons, the focus of this paper is on the MRI domain, aiming to provide a systematic overview of the radiomics harmonization strategies and to shed light on all challenges raised in every single step of the radiomics analysis workflow. For the convenience of the reader, this review study divides these approaches into two broad categories, although both approaches could also be used in conjunction: (i) the image-based harmonization techniques that are applied across images before the radiomics feature extraction, and (ii) the feature-based harmonization techniques, aiming to reduce the differences across the features by either modifying how these features are calculated or by post-processing them after their extraction [4,19]. Table 1 summarizes recent work on MRI radiomics harmonization.

## 2. Image-Based Harmonization Techniques

### 2.1. Image Acquisition and Reconstruction

In multicenter studies, scanner and protocol variability issues usually occur since diverse imaging data are collected from different clinical sites and acquisition protocols. Moreover, images are sensitive to inter-subject variations caused by the subject and the scanned body region. To confront this challenge, image acquisition and reconstruction parameters need to be standardized across the various centers that are responsible for the examined clinical study [19,48]. This can be achieved when issues regarding the scanner type, the acquisition and reconstruction parameters are equivalently considered in the initial design of the imaging protocol. It is of note to mention that in the case of CT and PET imaging, many studies investigated the impact of harmonized image reconstruction and acquisition parameters on feature consistency stating that harmonization on PET/CT images from different vendors can be achieved [49,50]. The European Association of Nuclear Medicine (EARL) program is commonly used to harmonize the systems, providing guidelines on scan acquisition, image processing, interpretation of images and patient preparation [51,52]. Additional guidelines, reviewed in [19], are provided by the European Society for Therapeutic Radiology and Oncology (ESTRO), the American Society for Radiation Oncology (ASTRO) and the Center for Drug Evaluation and Research (FDA). However, to the best of our knowledge, none of them refer to MRI [19].

### 2.2. Image Preprocessing

Image preprocessing is a significant part in the analysis pipeline that induces increased levels of variability in radiomics. It can be broken down into four consecutive steps: (i) interpolation, (ii) bias field correction, (iii) normalization, and (iv) discretization. Each step consists of various parameters with a considerable impact on the robustness and the absolute values of the features (e.g., distortions/non-uniformities attenuation due to noise, gray-scale and pixel size standardization) [2,20]. Indicatively, image preprocessing exhibited a significant impact in MRI studies when phantom [20] and glioblastoma data [21,53] were utilized to improve radiomics stability. From the technical perspective, image preprocessing is usually performed in computing environments like Python and Matlab and is often combined with software packages for radiomics features extraction (PyRadiomics, CERR, IBEX, MaZda and LIFEx) [2,48].

#### 2.2.1. Interpolation

Image interpolation, divided into upsampling and downsampling, manages to resize an image from an original pixel grid to an interpolated grid. Several interpolation algorithms are commonly used in the literature, including among others the nearest neighbor, trilinear, tricubic convolution and the tricubic spline interpolation. A thorough review is given in [54]. According to the image biomarker standardization initiative (IBSI) guidelines [54], interpolation is a prerequisite in radiomics studies since it enables texture features extraction from rotationally invariant three-dimensional (3D) images [54]. In addition, it ensures that spatially-related radiomics features (e.g., texture features) will be unbiased, especially in the case of MRI where images are often non-isotropic [2]. To this end, there is evidence that interpolating images in a consistent isotropic voxel space can potentially increase radiomics reproducibility in multicenter studies (e.g., CT and PET studies have shown dependencies between feature reproducibility and the selected interpolation algorithm [55,56,57,58]). This was also shown in [53] where three distinct measures assessed the impact of image resampling to reduce voxel size variations from images acquired at 1.5 T and 3 T; these measures were based on: (i) differences in feature distribution before and after resampling, (ii) a covariate shift metric, and (iii) overall survival prediction. To this end, isotropic resampling through a linear interpolation decreased the number of features dependent on the different magnetic field strengths from 80 to 59 out of 420 features, according to the two-sided Wilcoxon test. However, models deployed from the resampled images failed to discriminate between high and low overall survival (OS) risk (*p*-value = 0.132 using cox proportional hazards regression analysis). Phantom and brain MRI were isotropically resampled to voxels of length 1 mm in a study where combinations of several preprocessing and harmonization processes were applied to compensate for differences in the acquisition settings (magnetic field strength and image resolution) [22]. Radiomics reproducibility was evaluated on a feature-level using the “DiffFeatureRatio”, calculated as the ratio of the radiomics features with a *p*-value of less than 5% (different feature distributions due to scanner effects) to the overall radiomics features. In most of the cases, the isotropic voxel spacing caused a decrease in the “DiffFeatureRatio”, and a reduction of the scanner effect impact. However, still no clear recommendation can be made about the most effective interpolation technique in multicenter MRI radiomics [54]. In addition, although isotropic interpolation enables radiomics feature extraction in the 3D domain, a per slice (2D) radiomics analysis is recommended when slice thickness is significantly larger than the pixel size of the image (e.g., slice thickness of 5 mm and a pixel size between 0.5 and 1 mm) [6].

#### 2.2.2. Bias Field Correction

Bias field is a low frequency signal that may degrade the acquired image [59] and lead to an inhomogeneity effect across the acquired image. Its variation degree differs not only between clinical centers and vendors but also at the patient level even when a single vendor or acquisition protocol is used. Bias field correction can be implemented using Gradient Distribution Based methods [60], Expectation Maximization (EM) methods [61] and Fuzzy C-Means based [62]. However, N4 Bias Field Correction [63], an improved version of the N3 Bias Field Correction [64], has been one of the most successful and widely used techniques. It has been used extensively in various anatomical sites (e.g., brain tumor segmentation [65] and background parenchymal enhancement [66]) and the reason for its success is that it allows faster execution and a multiresolution scheme that leads to better convergence compared to N3 [63]. To this direction, a multicenter MRI study showed that when N4 bias field correction was applied prior to noise filtering, an increase in the total number of the reproducible features was achieved according to the concordance correlation coefficient (CCC), dynamic range (DR), and intra-class correlation coefficient metric (ICC) [21]. Indicatively, in the case of necrosis, the number of robust features was increased when radiomics was performed on the bias field corrected images rather than on the raw imaging data (32.7%, CCC and DR ≥ 0.9). Interestingly, when bias field correction was applied prior to noise filtering, the necrotic regions of the tumor had the highest number of extremely robust features (31.6%, CCC and DR ≥ 0.9). Another study explored stability of radiomics features with respect to variations in the image acquisition parameters (time of repetition and echo, voxel size, random noise and intensity of non-uniformity) [20]. MRI phantoms represented an averaging of 27 co-registered images of real patients (i.e., with different image acquisition parameters); and features with an ICC higher than 0.75 were reported as stable. The study showed that N4 Bias Field Correction, coupled with a common isotropic resolution resampling, had a significant impact on radiomics stability (particularly on first-order and textural features). Conclusively, Bias Field Correction is strongly recommended as a pre-processing step in multicenter studies.

#### 2.2.3. Intensity Normalization

To compensate for scanner-dependent and inter-subject variations, signal intensity normalization has been deployed to change the range of the signal intensity value within the ROI. This is achieved by calculating the mean and the standard deviation of the signal intensity gray-levels within the predefined ROI, or by transforming the ROI histogram to match a reference signal intensity 1-dimensional histogram [16,22,23,24,25,26,27,67]. The importance of intensity normalization was emphasized in the literature but no principal guidelines have been established yet. On the other hand, seven principles for image normalization (a.k.a SPIN) were proposed [68] in order to produce intensity values that: (i) have a common interpretation across regions within the same tissue type, (ii) are reproducible, (iii) maintain their rank, (iv) share similar distributions for the same ROI within and across subjects, (v) are not affected by biological abnormalities or population heterogeneity, (vi) are minimally sensitive to noise and artifacts, and (vii) do not lead to loss of information related to pathology or other phenomena. Noting that $I_{norm}\left(x\right)$ and $I\left(x\right)$ are the intensities of the normalized and the raw MRI respectively, the most commonly used intensity normalization techniques are outlined below (publicly available repositories are summarized in Table 2).

Z-score normalizes the original image $I\left(x\right)$ by centering the intensity distribution at a mean $\left(\mu \right)$ of 0 and a standard deviation $\left(\sigma \right)$ of 1 [68]. Computationally, Z-score is not time consuming and can be applied easily by subtracting the mean intensity either of the entire image or a specific ROI from each voxel value, followed by dividing the result by the corresponding standard deviation [26].

(1) $$I_{norm}\left(x\right)=\frac{I\left(x\right)-\mu }{\sigma }$$

WhiteStripe is a biologically driven normalization technique, initially deployed in brain radiomics studies, which applies a Z-score normalization based on the intensity values of the normal-appearing white matter (NAWM) region of the brain [68]. The NAWM is used as a reference tissue, since it is the most contiguous brain tissue and is, by definition, not affected by pathology (leading to conformity to SPIN 5) [68]. To this end, WhiteStripe normalizes the signal intensities by subtracting the mean intensity value of the NAWM $\left(\mu \right)$ from each signal intensity $I\left(x\right)$, and dividing the result by the standard deviation of the NAWM $\left(\sigma \right)$.

Min–Max standardizes the image by rescaling the range of values to [0, 1] using the Equation $\left(2\right)$, where the $min\left(x\right)\;\mathrm{and}\;max\left(x\right)\;$are the minimum and the maximum signal intensity values per patient, respectively [26].

(2) $$I_{norm}\left(x\right)=\frac{I\left(x\right)-min\left(x\right)}{max\left(x\right)-min\left(x\right)}$$

Normalization per healthy tissue population is performed when the signal intensity values of a given image are divided by the mean intensity value of the healthy tissue (e.g., adipose tissue or muscle in musculoskeletal imaging) [26].

(3) $$I_{norm}\left(x\right)=\frac{I\left(x\right)}{mean\left(x_{healtytissue}\right)}$$

Fuzzy C-means (FCM) uses fuzzy c-means to calculate a specified tissue mask (e.g., gray matter, white matter or the cerebrospinal fluid) of the image [27]. This mask is then used to normalize the entire image based on the mean value of this specified region. The method procedure is based on the following Equation $\left(4\right)$ where $c\in R_{>0}$ is a contrast that determines the specified tissue mean after normalization.

(4) $$I_{norm}\left(x\right)=\frac{c\;\cdot \;I\left(x\right)\;}{\mu }$$

Gaussian mixture model (GMM) assumes that: (i) a certain number of Gaussian distributions exist in the image, and (ii) each distribution represents a specific cluster [27]. Subsequently, GMM clusters together the signal intensities that belong to a single distribution. Specifically, GMM attempts to find a mixture of multi-dimensional Gaussian probability distributions that best model a histogram of signal intensities within a ROI. The mean of the mixture component, associated with the specified tissue region, is then used in the same way as the FCM-based method according to $\left(4\right)$ with a constant $c\in R_{>0}$.

Kernel Density Estimate (KDE) estimates the empirical probability density function (pdf) of the signal intensities of an image I over the specified mask using the kernel density estimation method [27]. The KDE of the pdf for the signal intensity of the image is then calculated as follows:

(5) $$\hat{p}\left(x\right)=\frac{1}{N\cdot M\cdot L\cdot \delta }\overset{Ν\cdot Μ\cdot L}{\underset{i=1}{\sum }}K\left(\frac{x-x_{i}}{\delta }\right),$$

where x is the intensity value, *K* is the kernel (usually a Gaussian kernel), and δ is the bandwidth parameter which scales the kernel *K*. The kernel density estimate provides a smooth version of the histogram which allows us to robustly pick the maxima associated with the reference mask via a peak finding algorithm. The peak ρ is then used to normalize the entire image, in the same way the FCM does. Specifically,

(6) $$I_{norm}\left(x\right)=\frac{c\;\cdot \;I\left(x\right)\;}{\rho }$$

where the $c\in R_{>0}$ is a constant that determines the reference mask peak after the normalization.

Histogram-matching is proposed by Nyul and Udupa to address the normalization problem by first learning a standard histogram for a set of images and then mapping the signal intensities of each image to this specific histogram [32,70]. The standard histogram learns through averaging pre-defined landmarks of interest (i.e., intensity percentiles at 1, 10, 20, …, 90, 99 percent [32]) of the training set. Then, the intensity values of the test images are mapped piecewise and linearly to the learned standards histogram along the landmarks.

Ravel (Removal of Artificial Voxel Effect by Linear regression) is a modification of WhiteStripe [69]. It attempts to improve the White Stripe by removing an unwanted technical variation, e.g., scanner effects. The Ravel normalized image is defined as:

(7) $$I_{ravel}\left(x\right)=I_{WS}\left(x\right)-\gamma_{x}{Ζ}^{Τ}$$

where $I_{WS}$ is the WhiteStripe normalized image, $\gamma_{x}{Ζ}^{Τ}$ represents the unknown technical variation and $\gamma_{x}$ are the coefficients of unknown variations associated with voxel *x*.

Several multicenter studies examined the influence of signal intensity normalization in MRI radiomics variability. Fortin et al. compared Ravel, Histogram Matching and WhiteStripe normalization methods using T1-weighted brain images [70]. Ravel had the best performance in distinguishing between mildly cognitively impaired and healthy subjects (area under the curve—AUC= 67%) compared to Histogram Matching (AUC = 63%) and WhiteStripe (AUC = 59%). Scalco et al. evaluated three different normalization techniques, applied to T2w-MRI before and after prostate cancer radiotherapy [43]. They reported that, based on the ICC metric, very few radiomics features were reproducible regardless of the selected normalization process. Specifically, first-order features were highly reproducible (ICC = 0.76) only when intensity normalization was performed using histogram-matching. A brain MRI study reported that Z-score normalization, followed by absolute discretization, yielded robust first-order features (ICC & CCC > 0.8) and increased performance in tumor grading prediction (accuracy = 0.82, 95% CI 0.80–0.85, *p*-value = 0.005) [16]. A radiomics study in head and neck cancer explored the intensity normalization effect in: (i) an heterogeneous multicenter cohort comprising images from various scanners and acquisition parameters, and (ii) a prospective trial derived from a single vendor with same acquisition parameters [26]. Statistically significant differences (according to Friedman and the Wilcoxon signed-rank test) in signal intensities before and after normalization were only observed in the multicenter cohort. Additionally, Z-Score (using ROI) and histogram matching performed significantly better than Min–Max and Z-Score, when the entire image was used. This indicates that the addition of a large background area to the Z-score calculations can adversely affect the normalization process. Intensity normalization should also be performed cautiously in cases where a healthy tissue is delineated as the reference ROI (e.g., WhiteStripe where Z-score normalization is based on the NAWM) since significant changes can occur in this area from pathological tissue changes and/or after treatment (e.g., structural and functional changes from a radiation therapy) that can potentially alter the signal intensity values within the reference ROI [26]. Summing up, to the best of our knowledge, there is no clear indication whether to use intensity normalization with or without a reference tissue. On the one hand, normalization like the Z-Score is simple to implement, as it requires only the voxels within the ROI. On the other hand, WhiteStripe and its modifications can potentially perform better when the reference ROI is accurately segmented and the corresponding areas is known to be unassociated with disease status and or other clinical covariates.

Deep Learning (DL) methods (Table 2) are also used, in lieu of the well-known interpolation methods for image normalization [4]. These methods rely on Generative Adversarial Networks (GAN) [37,41] and Style Transfer techniques (ST) [30,74]. When it comes to the GANs, the idea is to construct images with more similar properties so that the extracted radiomics features can be comparable. Despite their novelty, the phenomenon of disappearing gradients makes GAN training a challenging process because it slows down the learning process in the initial layers or even stops completely [19]. Furthermore, GANs are also prone to generate images with similar appearance as an effect of mode collapse which occurs when the generator produces only a limited or a single type of output to fool the discriminator [19]. Due to this, the discriminator does not learn to come out of this trap, resulting in a GAN failure. Last but not least, GAN-based models can also add unrealistic artifacts in the images.

A solution to this problem is Style Transfer where two images called Content Image (CI) and Style Image (SI) are used to create a new image that has the content of CI rendered according to the style of SI. This can help to overcome scanner acquisition and reconstruction parameter variability. The Style Transfer approach is used for image harmonization by either image-to-image translation or domain transformations [30] (Table 2), [74,75]. Although it can be achieved via Convolutional Neural Networks (CNNs) [76], there are other choices for style transfer such as GANs, used for PET–CT translation and MRI motion correction [42]. Due to the aforementioned disadvantages of GANs, which (in the case of vanishing gradients) can be unpredictable, the use of Style Transfer with CNNs is recommended.

#### 2.2.4. Discretization

Discretization, the last step of image preprocessing, aims to cluster the original signal intensities of the pixels according to specific range intervals. These intervals are the bins which compose the signal intensity histogram. The purpose of this step is to limit the range of the intensities in order to calculate the radiomics features more efficiently [77]. Discretization depends on the following parameters: (i) the range of the discretized quantity, and (ii) the number and the width of bins [2]. Fundamentally, the range equals the product of the bin number times the bin width. Thus, the crucial parameter of the discretization step is the proper choice of the number and the width of bins. However, optimal parameter choice has not been defined yet [2].

Recent studies have shown the impact of discretization on the reproducibility of MRI-based radiomics features [16,78,79,80,81,82]. Specifically, discretization had a direct impact on stability (irrespective of observers), on software and on segmentation methods [78]. As documented in related literature, the absolute discretization with fixed bin size/width (FBS) method and the relative discretization with fixed bin number (FBN) method are commonly used. According to the IBSI definition [54], the fixed bin size/width method consists of two different resampling methods outlined below:

(8) $$I_{BS}\left(i\right)=\left\{\frac{I\left(i\right)}{BS}\right\}-\left\{\frac{I_{min}}{BS}\right\}+1$$

where the $I_{BS}\left(i\right)$ is the discretized gray-level of the i-th voxel, $I\left(i\right)$ is the original i-th signal intensity, $I_{min}$ is the original minimum signal intensity of a particular ROI and BS is the bin width. The term $\left\{\frac{I_{min}}{BS}\right\}+1$ ensures that the gray-level rebinning starts at 1. Alternatively, the absolute resampling can be defined by:

(9) $$I_{BS}\left(i\right)=\left[\frac{I\left(i\right)}{BS}-1\right]$$

The relative discretization with fixed bin number is applied to every signal intensity from a pixel within the ROI to a fixed number of bins (*BN*) as defined by:

(10) $$I_{BN}\left(i\right)=\left\{\begin{array}{cc} \;\;\;1, & if\;\;I\left(i\right)=I_{min}\\ \left[BN*\frac{I\left(i\right)-I_{min}\left(i\right)}{I_{max}\left(i\right)-I_{min}\left(i\right)}\right], & otherwise \end{array}\right.$$

where $I_{BN}\left(i\right)$ is the discretized gray-level of the i-th pixel after FBN discretization, and *BN* corresponds to the fixed number of bins between $I_{min}$ and $I_{max}$, which are the minimum and the maximum intensities of the ROI, respectively.

Gray-level discretization using FBS was suggested in the literature since it provides: (i) more reproducible radiomics features compared to the FBN [78,80,82], and (ii) significantly lower ICC values when altering the bin size instead of changing the bin number of the FBN [78,79,80,81]. On this subject, Molina et al. reported variability issues in post-contrast T1-weighted MRI radiomics features of the brain using two different imaging protocols [79]. Specifically, their findings showed that texture features were highly sensitive to the bin number choice, further resulting in significantly different model results. Indicatively, in the case of lacrimal gland tumors and breast lesions, Duron et al. evaluated the inter- and intra-observer radiomics reproducibility using a combination of ICC and CCC metrics with 0.8 and 0.9 as thresholds, respectively [78]. When FBS and FBN were both applied in two independent MRI cohorts, high numbers of reproducible features were obtained using large bin numbers (i.e., 512, 1024) and small bin widths (i.e., 1, 5, 10, 20), respectively [78]. Moreover, they found that: (i) the FBS method provided a higher number of reproducible features than the FBN method; (ii) more consistent model results were reported from bin size variations than from bin number variation in FBN; and (iii) the FBS method is less sensitive to inter- and intra-observer segmentation variability. Two recent studies also showed radiomics features dependency on the binning parameters, stressing the importance for a careful optimal parameter selection in the discretization process [16,82]. Gergo Veres et al. reported the effect of two different FBS versions and of one FBN method in T1- and T2-weighted MRI from multiple sclerosis, ischemic stroke, and cancer [82]. Both FBS versions were favored by the analysis and recommended for discretization in brain MRI radiomics. Moreover, in brain MRI radiomics models, [16] also proposed, on one hand, the FBS method when using first- and second-order features, and on the other, the FBN when the models were constructed exclusively from second-order features. In both cases, 32 bins were recommended. It should be noted that although the FBN discretization is recommended for images with arbitrary units (e.g., T2 MRI) [54], recent research suggests that IBSI should explore the possibility of updating its guidelines to recommend the FBS discretization method in MRI studies [6,78]. For future multicenter studies, one common practice to investigate the impact of gray-level discretization on MRI could be the calculation of a scaling factor as shown in the equation below [16]:

(11) $$FBS=\frac{1}{FBN}\times mean_{Range}$$

where *FBS* and *FBN* correspond to the size and the number of bins, respectively; and $mean_{Range}$ is the mean of the intensity intervals of the region of interest.

### 2.3. Image Segmentation

Image segmentation is a standard in the radiomics analysis workflow with a considerable influence on the quality of the extracted features [2]. It can be performed by commercial-based and open-source tools, including among others the 3D Slicer [83] [slicer.org], MITK [mitk.org], ITK-SNAP [itksnap.org], MevISLab [mevislab.de], LifEx [lifexsoft.org], ImageJ [84] and in-house software [78]. Technically, image segmentation can be conducted manually, semi-automatically or fully automatically. In manual segmentation, the expected ROI is highly recommended to be drawn by domain experts since the observers’ expertise directly affects the repeatability and reproducibility of the radiomics features [78,85,86,87,88]. However, potential variations in their experience can still influence the reproducibility of the radiomics features. Semi-automatic segmentation is usually applied by region-growing algorithms [89,90,91], gradient-based models [92,93,94,95] or intensity thresholding-based methods [96], whereas deep learning algorithms nowadays achieve great performance in automated image segmentation [97,98,99,100].

Manual and semi-automated segmentations often suffer from the intra- and inter-observer segmentation variability, related to the degree of consistency between ROI delineations taken, whether by the same or different observers. The assessment is usually made by a feature stability analysis followed by selecting those particular features that are reproducible across the different ROI delineations. Indicatively, a study by [85] reported that shape features are more prone to inter-observer variability in MRI than in CT. While fully automatic techniques seem to be the best choice to eliminate observer variability, lack of generalizability limits their segmentation performance [2]. Loic Duron et al. conducted several experiments using manual (from three experts) and computer-aided annotations from two independent datasets [78]. A combined cutoff of pairwise ICC values from the manual annotations (>0.8) and CCC on the intra-observer pair (>0.9) was determined to select the most reproducible features. However, in most of the studies, intra and inter-observer segmentation variability is underestimated due to the specific number of readers (i.e., two readers) who perform the annotations [85,86,88]. The literature suggests that annotations should be delineated both from a fully automated segmentation tool and the clinical experts, followed by a Dice coefficient similarity score to assess agreement on the segmentations [78]. To this end, fully-automated segmentation using deep learning is nowadays becoming mainstream in radiomics studies. This includes the use of U-net on breast lesions [97], the DeepMedic and 3D CNNs on glioblastoma tumors [98,99] and the V-Net on prostate cancer [100]. However, it should be taken into consideration that generalizability in deep learning segmentation can only be achieved when a significant amount of data is used for training [2]. The ROI segmentation effect on different feature groups has been investigated extensively on PET/CT [101]. In the case of MRI, a radiomics variability study on cervical cancer found that entropy was the feature which was most reproducibly independent of observer effects (ICC > 0.9) [102], whereas Fiset et al. showed that shape features were the most stable ones in three different cohorts (ICC ≥ 0.75) when first-order, shape, texture, LoG and wavelet features were explored [103].

## 3. Feature-Based Harmonization Techniques

### 3.1. Batch Effect Reduction

#### 3.1.1. Combining Batches (ComBat)

ComBat is a data driven post-processing method and a widely used harmonization technique utilized to correct “batch-effects” in genomics studies [71]. Interestingly, it shows promising results towards facilitating features harmonization and reproducibility in radiomics studies [22,38,104]. From the technical perspective, it employs empirical Bayes methods to estimate the differences in feature values due to a batch effect. Subsequently, these estimates are used to adjust the data. ComBat performs location and scale adjustments of the radiomics features within centers in order to remove the discrepancies introduced by technical differences in the images [38]. Specifically, it assumes that feature values can be standardized by the following equation:

(12) $$Z_{ijg}=\frac{Y_{ijg}-\hat{a_{g}}-X\cdot \hat{\beta_{g}}}{\hat{\sigma_{g}}}$$

where *Ζ* corresponds to the standardized radiomics feature g for sample *j* and the center *I*; *Y* refers to a raw radiomics feature *g* for sample *j* and center *I*; $\hat{a\;}$ and $\hat{\sigma \;}$ are the features-wise mean; $\hat{\beta }$ is the standard deviation estimates; and *X* is a design matrix of non-center related covariates and is the vector of regression coefficients corresponding to each covariate. The standardized data is assumed to be normally distributed $Z_{ijg}\sim \;N\left(\gamma_{ig},\delta_{ig}^{2}\right)$, where γ and δ are the center effect parameters with Normal and Inverse Gamma prior distributions, respectively. Then, ComBat performs a feature transformation based on the empirical Bayes prior estimates for 𝛾 and 𝛿 of each center, respectively. The final center effect adjusted values (referred as $Y_{ijg}^{ComBat})\;$are given by:

(13) $$Y_{ijg}^{ComBat}=\frac{\hat{\sigma_{g}}}{{\hat{\delta }}_{ig}^{ComBat}}\cdot \left(Z_{ijg}-{\hat{\gamma }}_{ig}^{ComBat}\right)+\hat{a_{g}}-X\cdot {\hat{\beta }}_{g}$$

where ${\hat{\delta }}_{ig}^{ComBat}$ and ${\hat{\gamma }}_{ig}^{ComBat}$ are the Empirical Bayes estimates of ${\hat{\delta }}_{ig}^{2}$ and ${\hat{\gamma }}_{ig}^{}$, respectively. Recently, a comprehensive guide was provided on the proper use of Combat in multicenter studies [105].

Combat harmonization has been an essential part in radiomics analysis, especially in multicenter studies. In a recent study, ComBat contributed to facilitating radiomics harmonization when variability from cortical thickness measurements was evident in images acquired from 11 different scanners [28]. In [47], ComBat was enabled to transform MRI-based radiomics features derived from: (i) T1 phantom images and T1-weighted brain tumors using 1.5 T and 3 T scanners, and (ii) publicly available prostate data acquired in two centers. The statistical analysis (Friedman and Wilcoxon tests) demonstrated the elimination of the scanner effect and an increased number of statistically significant features to differentiate low- from intermediate/high-risk Gleason scores. In addition, a radiomics model based on linear discriminant analysis was implemented in order to distinguish low- from intermediate/high-risk Gleason scores. Before ComBat, the model yielded a Youden Index of 0.12 (Sensitivity = 19%, Specificity = 93%) while after ComBat, the Youden Index increased to 0.20 (Sensitivity = 27%, Specificity = 93%). Interestingly, when the Gleason grade was used as covariate, in ComBat the Youden Index increased to 0.43 (Sensitivity = 58%, Specificity = 86%). Another study [34] harmonized radiomics features from apparent diffusion coefficient (ADC) parametric maps and successfully predicted disease free survival and locoregional locally advanced cervical cancer when validated externally from two different cohorts (accuracy of 90%, 95% CI (79–98%), sensitivity 92–93%, and specificity 87–89%) after ComBat and (82–85%) without harmonization.

#### 3.1.2. M-ComBat

ComBat centers the radiomics features to an overall mean of all samples. Consequently, the new adjusted data are shifted to an arbitrary location that no longer concurs with the location of any of the original centers [38]. M-ComBat, a modified version of ComBat, addresses this problem towards shifting data to the mean and variance of the chosen reference center $\left(r\right)$. This is achieved by changing the standardized mean and variance of the estimates $\hat{a_{i}}$ and $\hat{\sigma_{i}}$ to center-wise estimates for $\hat{a_{i=r,g}}$ and $\hat{\sigma_{i=r,g}}\;$[88,89].

#### 3.1.3. B-ComBat, BM-ComBat

B-ComBat and BM-ComBat are two modifications of the initial models using bootstrapping. Initial estimates, obtained in ComBat and M-ComBat, are resampled B times with replacement. Resamples in both models are then fitted in order to obtain the B estimates of the coefficients ($\;\gamma_{ig}^{*},a_{g}^{*},a_{i=r,g}^{*},\delta_{ig}^{*}$). Finally, the estimates of the coefficients are computed using the Monte Carlo method by getting the mean of B estimates. In the literature, a notable increase in the performance of the radiomics models was reported when images were batch-effect corrected using either B-Combat or BM-Combat [38]. Specifically, Da-ano et al. assessed the impact of four different ComBat versions (ComBat, M-ComBat, B-ComBat, and BM-ComBat) when three radiomics models were deployed for locally advanced cervical cancer (LACC) and locally advanced laryngeal cancer (LALC) predictions. Although all versions succeeded in removing the differences among the radiomics features, it was M-ComBat and BM-ComBat that slightly outperformed other techniques in terms of model performance. Utilizing M-ComBat, balanced accuracy was increased from 79% to 85% for LACC and from 79% to 89% for LALC. An increase in the performance was also evident in the case of BM-ComBat (i.e., balanced accuracy was increased from 82% to 89% for LACC and from 82% to 86% for LALC).

#### 3.1.4. Transfer Learning ComBat

One of the basic limitations of ComBat is its inability to harmonize new “unseen data”, thus images, acquired from a center or a vendor never seen at the feature transformation phase. In other words, once new data from a different source is added in the analysis, ComBat needs to re-establish and apply from scratch the harmonization process to the updated cohort in addition to the new images. To address this issue, a study in [44] proposed a modified ComBat coupled with a transfer learning technique to permit the use of previously learned harmonization transforms to the radiomics features of the “unseen data”. This integration was performed on the original ComBat and all of its modifications with satisfactory results.

#### 3.1.5. Nested ComBat, NestedD ComBat

Nested ComBat, differentiating from the original version where radiomics features are harmonized by a single batch effect at a time, provides a sequential radiomics features harmonization workflow to compensate for multicenter heterogeneity caused by the multiple batch effects [72]. In addition, Horng et al. claimed that features may not only follow a Gaussian distribution, as ComBat assumes, but a bimodal distribution as well [72]. To handle this, the authors introduced NestedD, with the purpose of the evaluation of their hypothesis that removing significantly different features in their distributions across the batch effects could potentially improve harmonization performance. Although this process is contrary to ComBat methodology which exploits all available information [38], NestedD was recommended for high-dimensional datasets where there is room for improvement towards reducing the information loss effect [72]. It is worth mentioning that the performance of both alternatives is not yet evaluated in MRI studies. Both methods are publicly available as referred to in Table 2.

#### 3.1.6. GMM ComBat

The Gaussian Mixture Model (GMM) is another ComBat modification, relying on the GMM split method to handle bimodality coming from the variation of unknown factors [72]. It comes as an alternative to ComBat to confront potential issues arising from the assumption that all imaging factors (batch effects and clinical covariates) become known in light of the variation in results due to new factors and that cannot be corrected. To the best of our knowledge, GMM ComBat has so far only been evaluated on CT studies.

#### 3.1.7. Longitudinal ComBat

Although longitudinal (or temporal) data are important for the measurement of intra-subject change, previous ComBat variants focus exclusively on radiomics harmonization from cross-sectional data. To further expand ComBat applicability in the longitudinal domain, authors from [29] used the following mixed linear:

(14) $$y_{ijv}\left(t_{k\;}\right)=a_{v}+\beta_{v1}\left(baseline\;age\right)+\beta_{v2}\;I\left(sex=male\right)+\beta_{v3}I\left(diagnosi=LMCI\right)+\beta_{v4}\;I\left(diagnosis=AD\right)\;+\beta_{v5}\cdot t_{k\;}+\beta_{v6}\;I\left(diagnosis=LMCI\right)\cdot t_{k}+\beta_{v7}I\left(diagnosis=AD\right)\cdot t_{k\;}+\beta_{v8}I\left(scanner_{k\;}=2\right)+\cdots +\beta_{v\left(m+7\right)}I\left(scanner_{k\;}=m\right)+\eta_{jv}+\varepsilon_{ijv}\left(t_{k\;}\right)$$

where the extra insertions are the additive terms of the factor $t_{k}$, the number of years that are passed from baseline age, and $I\left(.\right)$ is an indicator function equal to one if the argument condition is true and zero otherwise. Hence, the longitudinally-oriented ComBat (source code is available on GitHub, Table 2) achieves the removal of additive and multiplicative scanner effects to such a sufficient extent that no scanner covariates are prerequisite in the final implementation. Public implementation of the method is referenced in Table 2.

### 3.2. Deep Learning

Deep Learning is an alternative to the batch effect removal methods described above. Specifically, Andrearczyk [106] proposed a deep-learning model that learns a non-linear transformation for the normalization of radiomics features, both in handcrafted and deep features. In [107] and [74]], a two-stream CNN architecture with unshared weights and the DeepCORAL [108] were used in order to reduce divergence between source and target data feature distributions (both use non-medical images) [19]. Although successful with non-medical images, these methods tend to be less successful with medical imaging data. The approach adopted in [46] was a Domain Adversarial Neural Network that constructed an iterative update approach, aiming to generate scanner-invariant (i.e., harmonized features) representations of MRI neuroimages. The method of [46], having been tested on a multi-centric dataset, seems a more reasonable approach for feature harmonization.

### 3.3. Feature Extraction and Reduction/Selection

When different software packages are used, it is evident that computational differences in the radiomics extraction not only produce increased variability in the feature values but also influence the reliability and the prognostication ability of the radiomics models. Although this specific part of the analysis is far beyond the scope of this review, readers should be aware of these challenges, investigated thoroughly in [109,110,111]. Discarding or selecting radiomics features based on their reproducibility is another crucial step to enable the construction of robust radiomics models [4]. The challenge here is to find the ideal threshold that will eventually select the stable radiomics features with respect to the variability issues prior to their reduction. Many studies have investigated the radiomics features reproducibility across scanners and protocols, usually using the concordance correlation coefficient (CCC) and the Intraclass Correlation Coefficient (ICC) [112,113,114]. However, these approaches do not guarantee their discriminative power [19].

## 4. Discussion

Variations derived from different scanner models, reconstruction algorithms, acquisition protocols and image preprocessing, followed by feature extraction, are frequently unavoidable phenomena in multicenter radiomics studies. Hence, radiomics harmonization has become an integral part in all analysis steps, since only robust and stable features can potentially enable reproducibility and generalizability in radiomics modeling. To address these issues, several studies examined harmonization across different sites and scanners; nevertheless, radiomics variability still remains under investigation [29,115]. Another effort from the IBSI focused on the standardization of the image preprocessing and the radiomics feature extraction phase; however, little attention has been paid to MRI, compared to CT and PET studies [54]. Indicatively, in the imaging preprocessing phase, still no clear recommendation can be made about the most effective interpolation technique or the appropriate size of bin/width in the discretization step. Nevertheless, N4 Bias Field Correction and isotropic interpolation are frequently used in MRI radiomics. In addition, there are different opportunities and limitations related to choosing an intensity-based or a feature-based harmonization technique in terms of restrictions on the number of samples collected for the analysis. For instance, the ComBat method requires a minimum number of 20–30 patients per batch [86] in contrast to the Z-score or White-Stripe normalization method.

To this end, harmonization strategies were utilized and guidelines were proposed to reduce the unwanted “center-effect” variation, mostly focused on CT and PET studies [19]. Regarding MRI, UCHealth made an effort to reduce the number of MRI protocols from 168 to 66 across scanners and centers, aiming to provide a suitable clinical-driven protocol and standardization approach [19]. In addition, the Prostate Imaging Reporting and Data System (PIRADS) guidelines, currently still in progress, put efforts in the context of image acquisition standardization and prostate MRI interpretation [6]. They stated that in order to potentially compensate for the various combinations of the protocol parameters, there is an urgent need to standardize MRI acquisition protocols according to specific guidelines.

The review highlights both the impact of image preprocessing and feature-based harmonization methods in radiomics reproducibility among different scanners and protocols. However, very little can be found in the literature concerning comparisons between intensity and feature-based harmonization techniques. Recently, Li et al. investigated potential reductions in MRI scanner effects and increased reproducibility when image preprocessing (N4 bias field correction and image resampling) and harmonization (intensity normalization and ComBat) where applied to: (i) homogeneous and heterogeneous phantom data, and (ii) brain images from a healthy population and from patients with tumor [22]. On the one hand, ComBat succeeded in removing scanner effects (“DiffFeatureRatio” tended to zero in all cases). On the other hand, none of the Z-score, WhiteStripe, FCM, GMM, KDE and histogram-matching succeeded in improving crucial radiomics reproducibility, further indicating that intensity normalization might not suffice for radiomics harmonization. This was also mentioned in other studies where both intensity-normalization and feature-based harmonization were used in conjunction (e.g., using intensity normalization and ComBat) [22,35,45]. In other recent work [24], a cohort comprising T2-weighted MR images from patients with sarcoma was used to assess the influence of four different intensity-normalization techniques and ComBat on metastatic relapse at 2-years prediction. It was documented that each method had a different impact on model predictions. Overall, however, ComBat supplied the most realistic and concise results from both the training and the testing phase of the models. Although far beyond the scope of this review study, it is worth mentioning that publicly available machine learning (ML) based harmonization tools are currently distributed online. NeuroHarmony (Table 2) [73] is an indicative example, capable of providing harmonization for a single image despite having no knowledge of the image acquisition specifications.

Since deep learning has long been used for imaging, it is not surprising that it found its way in to radiomics harmonization. The key to its success is the ability to detect nonlinear patterns between features [116]. In [40], cycle-consistent GANs used 2 generator-discriminator pairs to achieve harmonization of breast dynamic contrast enhanced (DCE)-MRI. Moreover, a dual-GAN outperformed more conventional approaches such as voxel-wise scaling and ComBat when applied to diffusion tensor imaging (DTI) data [41]. Last but not least, in [39] and [31], auto-encoding methods and cycle-consistent adversarial networks (DUNCAN) were developed respectively to compensate for intersite and intersubjective variability effects. The most well-known application of DL in image-based harmonization is DeepHarmony [117], an enhanced version of NeuroHarmony, which uses a full-CNN based on U-Net for contrast harmonization. Compared to other methods, it improved volume correspondence and stability in longitudinal segmentation. Specifically, longitudinal MRI data of patients with multiple sclerosis were used to evaluate the effect of a protocol change on atrophy calculations in a clinical research setting [117]. Compared to the results obtained from other state-of-the-art techniques, the DeepHarmony images were less affected by the protocol change.

Other important issues that also need to be taken into account are the various ethical and legal constraints as well as organizational and technical obstacles that make sharing data between institutions a difficult process [118]. In [118], the Joint Imaging Platform (JIP) of the German Cancer Consortium (DKTK) addresses these issues by providing federated data analysis technology in a secure and compliant way. Via JIP, medical image data remain in the originator institutions, but analysis and AI algorithms are distributed and jointly used across the stakeholders. Radiomics can change the daily clinical practice, especially when combined with genomic and clinical information, since it provides clinicians with a predictive/prognostic non-invasive method [119]. Furthermore, medical data can be investigated rapidly [119]. However, as noted in this review, radiomics features yield a high rate of redundancy, i.e., from a large number of extracted features only a small number are truly useful, making even more challenging the decision over which radiomics are useful for clinicians to make an accurate diagnosis [54,120,121].

## 5. Conclusions

Radiomics provides great promise in digital diagnostics for more precise diagnosis, therapy planning, and disease monitoring as long as it is accompanied by a properly selected harmonization strategy to account for its inherent variability. This review finds that harmonization is not a black-box solution and has to be considered as a “hyperparameter” of the radiomics analysis pipeline, since dependencies on unstable parameters emanate from all radiomics analysis steps described above. To this end, the review gives an overview of several harmonization methods that are applicable to every single radiomics analysis phase, and highlights that all techniques should be considered and investigated both in terms of increasing radiomics reproducibility and optimizing model performance. This is especially recommended in MRI, which is characterized by an absence of a standard intensity scale and well-defined units. Indeed, a lot of work is still required to define comprehensive and MRI specific guidelines for radiomics harmonization in multicenter studies. We strongly support the formulation of a standardized acquisition protocol which still remains the cornerstone for improving the reproducibility of radiomics features, and the definition of a very specific reporting guideline (e.g., IBSI) in radiomics analysis. However, standardized guidelines should be constantly updated to keep pace with the rapid development of radiomics modeling techniques. The message that this review seeks to convey is that unstable and non-reproducible radiomics features along with inadequate harmonization techniques can potentially limit the discovery of novel image biomarkers from multicenter radiomics studies, subsequently hampering the trustworthiness and adoption of such approaches in the real clinical setting.

## Figures and Tables

**Table 1:** Radiomics harmonization in recent multicenter MRI studies.

Reference	MRI Sequences/ Cancer Type	Variation Across	Harmonization Techniques	Clinical Question
A. Carre et al. (2020) [16]	T1w, T2w-fl1air (brain-glioma)	3 centers	Intensity-based	Tumor grade classification
M. Bologna et al. (2019) [20]	T1w, T2w (brain)	27 centers	Intensity-based	Feature stability
H. Moradmand et al. (2019) [21]	FLAIR, T1, T1C, and T2 (brain-glioblastoma)	8 centers	Intensity-based	Edema, necrosis, enhancement, and tumor
Y. Li et al. (2021) [22]	T1 (brain)	2 scanners	Both	Scanner effect removal
L. J Isaksson et al. (2020) [23]	T2w (prostate cancer)	1 scanner	Intensity-based	Cancer identification
A. Crombe et al. (2020) [24]	T2w (sarcoma)	3 centers	Intensity-based	Prediction of metastatic-relapse-free survival
Chatterjee et al. (2019) [25]	T2-weighted fast spin echo (denoted as T2); T1-weighted fast gradient echo with DCE-MRI and a postcontrast image (post-Gado); and diffusion-weighted MRI (primary uterine adenocarcinoma)	2 centers	Intensity-based	Lymphovascular space invasion and cancer staging
K.A. Wahid et al. (2021) [26]	T2w (head and neck)	15 HET cohorts-15 HOM- cohorts	Intensity-based	Radiotherapy treatment
J. C. Reinhold et al. (2018) [27]	T1-w, T2-w, and FLAIR (brain)	1 dataset with 18 patients	Intensity-based	Medical image synthesis
J. P. Fortin et al. (2018) [28]	MRI (brain)	11 scanners	Feature-based	Cortical thickness harmonization
J. C. Beer et al. (2019) [29]	Structural MRI (brain)	58 sites	Feature-based	Alzheimer prediction
C. Ma et al. (2019) [30]	3D Cardiac MRI	20 scans (10 training and 10 test)	Intensity-based	Image segmentation
D. Tian et al. (2022) [31]	T1 MRI (brain)	12 centers	Intensity-based	Gray matter analysis
M. Shah et al. (2011) [32]	T1w, T2w, and PDw (brain)	10 scanners	Intensity-based	Multiple sclerosis lesion identification
Liu et al. (2018) [33]	T2w (brain-glioma)	2 cohorts	Intensity-based	Prediction of progression-free survival in lower-grade gliomas
F. Lucia et al. (2019 [34])	T1, T2 DWI (cervical cancer)	3 centers	Feature-based	Cervical cancer prognosis
Peeken et al. (2019) [35]	Contrast-enhanced T1-weighted fat saturated (T1FSGd), fat-saturated T2-weighted (T2FS) (sarcoma)	2 cohorts	Intensity/ feature-based	Classification of low and high grade soft tissue sarcoma
Liu et al. (2019) [36]	T1w,T2w-fl1air (brain)	4 centers	Feature-based	Prediction of the individualized treatment response in children with cerebral palsy
C. Hognon et al. (2019) [37]	T1, T1c, T2, FLAIR (glioblastoma)	3 centers	Intensity-based	Image segmentation
R. Da-Ano et al. (2020) [38]	Post-injection gadolinium contrast-enhanced MRI (GADO), T2-weighted MRI (T2) and apparent diffusion coefficients (ADC) maps from diffusion-weighted MRI (cervical cancer)	3 centers	Feature-based	Prediction and treatment adaptation
D. Moyer et al. (2020) [39]	Diffusion MRI (brain)	15 patients from 2 scanners	Intensity-based	White Matter analysis
G. Modanwal et al. (2020) [40]	DCE-MRI (breast cancer)	124 patients from 2 scanners	Intensity-based	Evaluation
J. Zhong et al. (2020) [41]	Neonatal DTI-MRI (brain)	84 neonates data from 2 sites	Deep Learning	Harmonize neonatal data
K. Armanious et al. (2020) [42]	T1 (brain)	11 patients	Intensity-based	Motion correction
Scalco et al. (2020) [43]	T1w, T2w (prostate)	3 different organs of interest	Intensity-based	Reproducibility estimation
R. Da-Ano et al. (2021) [44]	Post-injection gadolinium contrast-enhanced MRI (GADO), T2-weighted MRI (T2) and apparent diffusion coefficients (ADC) maps from diffusion-weighted MRI (cervical cancer)	3 centers	Feature-based	Prediction
Saint Martin et al. (2021) [45]	T1, T2, T1-DCE (breast)	2 phantoms (2 scanners and 3 dual breast coils)	Both	Lesion classification
N.K. Dinsdale et al. (2021) [46]	T1w (neuroimages)	3 dataset centers	Feature-based	Age prediction and segmentation
F. Orlhac et al. (2021) [47]	T1, FLAIR and contrast-enhanced T1-weighted (CE-T1w) images and T2w (brain/prostate)	2 centers	Feature-based	Impact of harmonization to distinguish between Gleason grades

**Table 2:** Harmonization techniques publicly available on GitHub.

Reference.	Harmonization Method	GitHub
Reinhold et al. [27]	Fuzzy C-means	https://github.com/jcreinhold/intensity-normalization, accessed on 9 September 2022
Reinhold et al. [27]	Gaussian mixture model (GMM)	https://github.com/jcreinhold/intensity-normalization, accessed on 11 October 2022
Reinhold et al. [27]	Kernel Density Estimate (KDE)	https://github.com/jcreinhold/intensity-normalization, accessed on 30 August 2022
Shinohara et al. [68]	Z-Score	https://github.com/jcreinhold/intensity-normalization, accessed on 8 October 2022; https://github.com/Jfortin1/RAVEL, accessed on 8 October 2022
Shinohara et al. [68]	White Stripe	https://github.com/jcreinhold/intensity-normalization, accessed on 11 September 2022; https://github.com/Jfortin1/RAVEL, accessed on 11 September 2022
Fortin et al. [69]	Ravel	https://github.com/jcreinhold/intensity-normalization, accessed on 12 August 2022; https://github.com/Jfortin1/RAVEL, accessed on 12 August 2022
Nyul et al. [70]	Histogram Matching	https://github.com/jcreinhold/intensity-normalization, accessed on 24 October 2022; https://github.com/Jfortin1/RAVEL, accessed on 24 October 2022; https://github.com/sergivalverde/MRI_intensity_normalization, accessed on 24 October 2022
Johnson et al. [71] Fortin et al. [28]	ComBat, M-Combat	https://github.com/Jfortin1/ComBatHarmonization, accessed on 18 September 2022; https://github.com/ncullen93/neuroCombat/tree/master/neuroCombat, accessed on 18 September 2022
Horng et al. [72]	Nested & GMM ComBat	https://github.com/hannah-horng/generalized-combat, accessed on 3 October 2022
Beer et al. [29]	Longitudinal ComBat	https://github.com/jcbeer/longCombat, accessed on 31 October 2022
Johnson et al. [71] Fortin et al. [28]	ComBaTool Standalone web application	https://forlhac.shinyapps.io/Shiny_ComBat/, accessed on 27 September 2022
Garcia-Dias et al. [73]	NeuroHarmony	https://github.com/garciadias/Neuroharmony, accessed on 23 August 2022
C. Ma et al. [30]	Deep Learning	https://github.com/horsepurve/StyleSegor, accessed on 21 September 2022
D. Tian et al. [31]	Deep Learning	https://github.com/DezhengTian/DeRed-Harmonization, accessed on 22 September 2022

## Data Availability

Not applicable.
